# Applying ^1^H NMR Spectroscopy to Detect Changes in the Urinary Metabolite Levels of Chinese Half-Pipe Snowboarders after Different Exercises

**DOI:** 10.1155/2015/315217

**Published:** 2015-05-25

**Authors:** Fuqiu Wang, Jiao Han, Qing He, Zhufeng Geng, Zhiwei Deng, Decai Qiao

**Affiliations:** ^1^College of P.E and Sports, Beijing Normal University, No. 19 Xinjiekouwai Street, Haidian District, Beijing 100875, China; ^2^Center of Analysis and Test, Beijing Normal University, No. 19 Xinjiekouwai Street, Haidian District, Beijing 100875, China; ^3^School of Chemical Engineering and Technology, Tianjin University, 92 Weijin Road, Nankai District, Tianjin 300072, China

## Abstract

Monitoring physical training is important for the health and performance of athletes, and real-time assessment of fatigue is crucial to improve training efficiency. The relationship between key biomarkers and exercise has been reported. The aim of this study was to determine the effects of different levels of training exercises on the urine metabolome. ^1^H NMR-based metabolomics analysis was performed on urine samples from half-pipe snowboarders, and spectral profiles were subjected to PCA and PLS-DA. Our results show that metabolic profiles varied during different stages of exercises. Lactate, alanine, trimethylamine, malonate, taurine, and glycine levels decreased while TMAO and phenylalanine levels increased in the stage with higher amount and intensity of exercise. Although the amount of exercise was reduced in subsequent stage, no significant variations of metabolic profile were found. Metabolic changes induced by training level were analyzed with related metabolic pathway. Studying metabolome changes can provide a better understanding of the physiology of athletes and could aid in adjusting training.

## 1. Introduction

The aim of physical training is to enhance athletic performance and prevent serious injury. Normally, physical training is often associated with a type of tissue trauma called “adaptive microtrauma” [1]. With adequate recovery, the body adapts to the training. Helping the body adapt to higher-intensity stimuli, therefore, is important for physical training [2, 3]. However, overtraining can lead to overtraining syndrome, which is often accompanied by physiological, biochemical, immunological, and psychological symptoms. Margonis et al. [4] found that 37% of professional athletes in all types of sports have suffered from overtraining syndrome at least once in their careers. Hence, monitoring physical training is essential. Many studies indicate that exercises stimulate the body producing changes in the metabolite levels of athletes, regardless of whether the exercise follow acute or chronic, planned or unplanned protocols [5, 6], even in strength-endurance sports [3]. Blood and urine were usually selected as indicators in monitor procedure. Previous studies utilized invasive methods and focused on single diagnostic metabolites, like lactate, phosphocreatine, or certain hormones [7]. However, variations in some of these indicators were not observed through detection of target biochemicals such as serum cortisol, testosterone, or creatine kinase [3].

Following the development of “omics” techniques, the use of metabonomics has increased rapidly in the past decade. As Nicholson et al. [8] stated, metabonomics is a measurement of the dynamic metabolic response of living systems to stimuli or modification. Metabonomics approaches can detect changes in the levels of many small molecules that arise as a response to cells or an organism to endogenous or exogenous stimuli, such as disease, development, environmental changes, or nutrient availability [9].

Metabonomics technologies have already been applied in the physiological monitoring of human movement [10]. However, studies applying metabonomics in sports are not numerous [7]. The reason is that there are many technical barriers, such as identifying plenty of metabolites and interpreting acquired data through advanced multivariate statistical analysis [9]. Hence, metabolomics is conducted by using sensitive analytical techniques, such as liquid chromatography-mass spectrometry (LC-MS), gas chromatography-mass spectrometry (GC-MS), and nuclear magnetic resonance (NMR) spectroscopy [11]. To date, many studies have analyzed metabolite profiles or conducted metabolite fingerprinting. Recent metabolomics studies of physical training have focused on blood serum or plasma in human and animal models. These studies have studied the effects of strenuous endurance exercises [12], strength-endurance training [3], specific diets [13], or ingestion of different beverages after exercise [14–17]. The effects of acute and chronic exercise and different types of exercise on the human urine metabolome have also been studied [6, 18]. Several studies have used saliva to assess the levels of metabolites in response to exercise and training or to evaluate physical performance [7, 19]. Enea et al. [18] detected changes in the concentration of 11 metabolites after exercise by using ^1^H NMR analysis of urine from female athletes and nonathletes.

Generally, previous studies have shown that exercise with a variety of forms and levels could cause metabolic changes in the body's biological fluids, such as blood, urine, and saliva. The test subjects in majority of studies were under strict requirements (diets, fasting, specific training plans with rest intervals, etc.) to obtain variations of key metabolites, aiming for hypotheses testing regarding specific biochemical pathways [6]. However, under actual circumstances, athletic training follows schedules, which was a combination of exercises in various forms. It is important to control the amount and intensity of physical exercise so as to obtain positive effects without fatigue. Thus, the present study uses traditional monitoring methods and NMR-based metabonomics to determine changes in the levels of small-molecule metabolites in urine during training process.

We describe nontargeted ^1^H NMR-based metabonomics analyses of urine samples obtained from professional snowboarders after weekly physical training. The aim of this work was to explore how physical exercise broadly impacts and changes metabolite profiles. Furthermore, we determined the feasibility of using metabonomics to assess and monitor periodic physical exercise.

## 2. Experimental Section

### 2.1. Sample Collection

**Figure 1:**
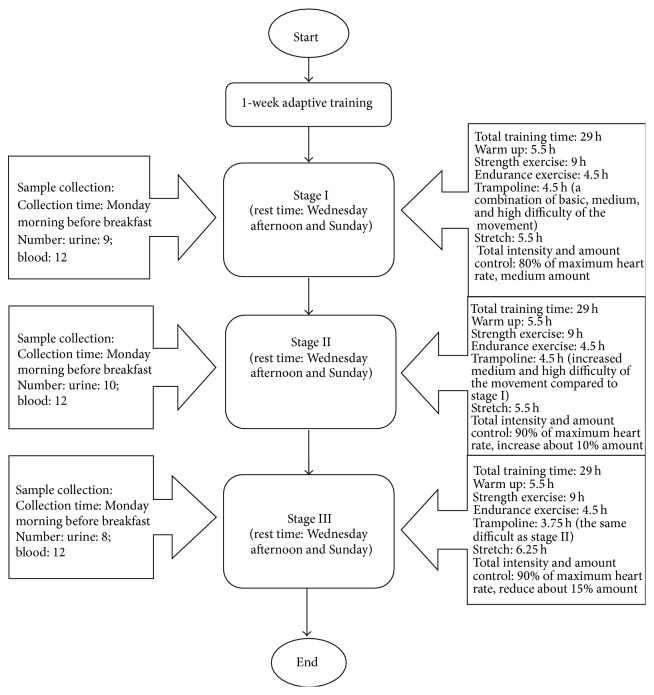
The flowchart for illustration of three-stage/level training exercises.

Twelve professional half-pipe snowboarders between the ages of 21–25 years with an average Body Mass Index (BMI) of 21.7 ± 1.5 kg/m^2^ participated in this study. All athletes had a standard diet and none used pharmaceutical products or tobacco during the training period. Physical training consisted of typical waist and abdominal strength and endurance and trampoline exercises, which were divided into three stages (stages I, II, and III; see Figure 1). Before the three-week training, subjects underwent a one-week adaptive training period. One training stage consisted of 5.5 days of training and 1.5 days of rest. All urine and blood samples were collected before breakfast on Monday morning. At the beginning of formal training, subjects completed strength, endurance, and trampoline exercises of a predefined level for one week. The next week, the amount and intensity of exercise were both increased by about 10% (warm up and stretch are not included in intensity calculation). Since there is an individual difference in the athletes, especially the gender difference, the changes of training level were quantitatively estimated according to the heart rate of athletes and the professional coach's personalized training. During the third week, the training intensity is the same as the second week, but the amount of exercise was reduced by about 15%. Nine urine samples were collected for stage I, and ten samples were collected for stage II. Only eight urine samples were collected for stage III. Blood samples were collected for all three stages. Urine samples were stored at −20°C with sodium azide until analysis.

### 2.2. Sample Preparation and Clinical Data Collection

Urine samples were thawed and centrifuged at 13,000 ×g for 10 min. Supernatant aliquots of 300 *μ*L were transferred to standard 5 mm NMR tubes and mixed with 300 *μ*L phosphate buffer (pH 7.4) and 50 *μ*L D_2_O containing sodium 3-(trimethylsilyl)-1-propanesulfonate (DSS, 0.1% w/v in D_2_O).

Serum cortisol and testosterone concentrations were measured by automatic biochemical analyzer. CK concentration was measured using diagnostic kits. BUN was measured by the urease UV method. Routine blood tests were used to analyze WBC, LYM, MXD, NEUT, RBC, HGB, HCT, MCV, and PLT.

### 2.3. ^1^H NMR Spectroscopy of Urine Samples

**Figure 2:**
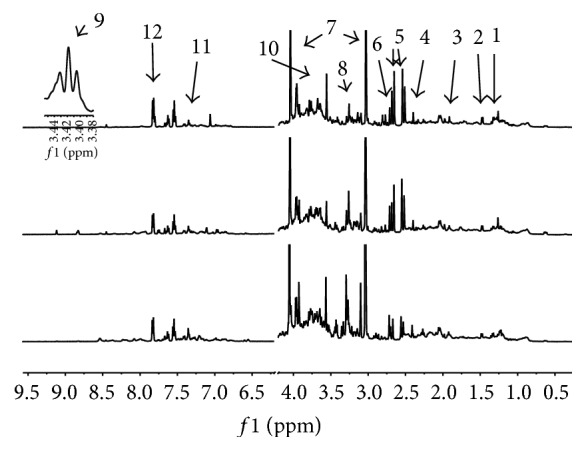
Typical 500 MHz 1D-NOESY NMR spectra of the urine sample from an athlete. (1) Lactate (d, 1.32 ppm); (2) alanine (d, 1.47 ppm); (3) acetate (s, 1.91 ppm); (4) succinate (s, 2.39 ppm); (5) citrate (dd, 2.51, 2.54, 2.65, and 2.68 ppm); (6) dimethylamine (s, 2.71 ppm); (7) creatinine (s, 3.03 ppm, s, 4.04 ppm); (8) TMAO (s, 3.26 ppm); (9) taurine (t, 3.26 ppm, t, 3.42 ppm); (10) glycine (s, 3.55 ppm); (11) phenylalanine (m, 7.42 ppm); (12) hippurate (d, 7.82 ppm).

^1^H NMR acquisition was performed using a Bruker DRX-500 spectrometer equipped with a BBFO probe with *z*-axis gradients maintained at 298 K. One-dimensional NMR spectra were recorded using a standard 1D NOESY pulse sequence with water suppression. A total of 256 scans were collected in 32 K data points with a spectral width of 8012.8 Hz. The acquisition time was 2.04 s, and relaxation delay was 2 s. All spectra were processed using MestReNova software (v. 8.1.2; Mestrelab Research SL, Santiago de Compostela, Spain). Spectra were manually referenced to the chemical shift of DSS before manual phase and baseline correction. On the purpose of identification, DQF-COSY and HSQC spectrum of representative sample were acquired (see Figures 1S, 2S, and 3S in Supplementary Material available online at http://dx.doi.org/10.1155/2015/315217).

### 2.4. Statistical Analysis

The spectral region of *δ* 0.5–10 ppm of all spectra was segmented into 0.02 ppm portions, excluding the 4.2–6.2 ppm region. Signal intensity was then normalized to the total spectral area and imported into Matlab (R2010a; Mathworks, Inc., Natick, MA, USA).

Multivariate data analysis of NMR data was performed using PLS Toolbox (Eigenvector Research, Inc., Manson, WA, USA). A principal component analysis (PCA) model was applied to determine the distribution and separation trends. To enlarge the difference and determine which variables contribute to separation, a partial least squares projection to latent structures and discriminant analysis (PLS-DA) model were then constructed. Cross-validation was conducted to ascertain the predictive ability of the model using the leave-one-out method. *R* ^2^ represents the variance captured by the model and *Q* ^2^ represents the prediction ability of the model. Furthermore, a permutation test was applied to validate whether models were overfit.

To identify important biomarkers, the clinical parameters of three groups were analyzed by Student's *t*-test. A nonparametric version of one-way ANOVA test [20] was also performed on NMR data of identified metabolites. The statistical significance level was set at *P* = 0.05.

Hierarchical clustering analysis [21] was used to assemble all variables into a single hierarchical tree and cluster relatively homogeneous elements together. In this study, we generated dendrograms and heat maps in Matlab based on measured data.

## 3. Results

### 3.1. Clinical Data

There were no significant differences in the concentrations of testosterone, cortisol, creatine kinase, BUN, WBC, LYM, or NEUT between the first two stages. The concentrations of MXD, RBC, MCV, and PLT between the first two stages were significantly different, as were the HGB values in samples from male individuals. However, most of the clinical parameters, such as MXD and RBC did not significantly differ between the second and third groups. MCV, PLT, and HGB concentrations from male individuals reached levels of statistical significance in the last two groups. Comparing the levels of MCV, PLT, and HGB (M) in these three groups, the HGB (M) level rose in the second stage and decreased in the third stage, whereas MCV and PLT values showed decreased levels in the second stage with higher levels approaching initial values in the third stage (Table 1).

### 3.2. ^1^H NMR Spectra and Metabolic Changes

**Figure 3:**
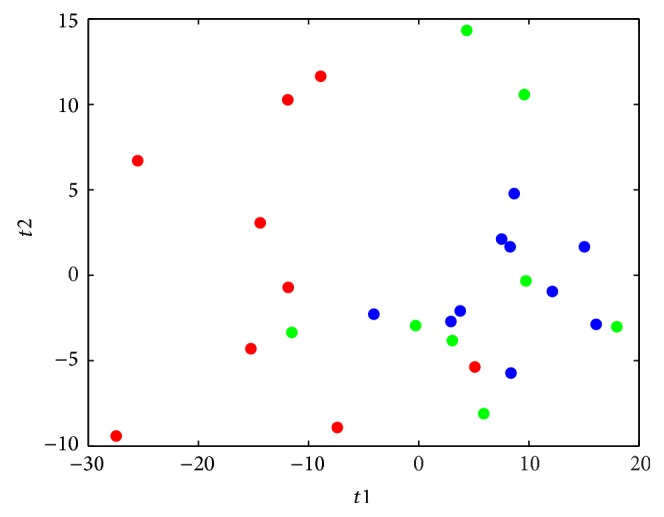
PCA score plot of NMR data from three stages (stage I: red dots; stage II: blue dots; stage III: green dots).

**Figure 4:**
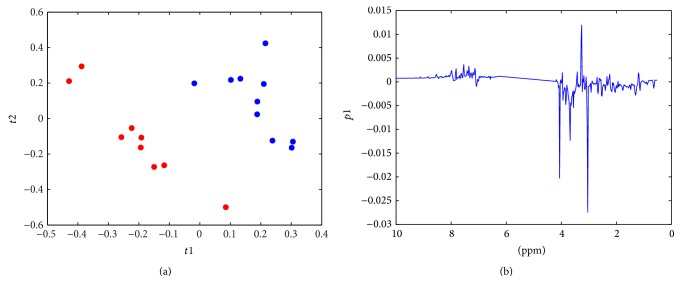
(a) Score plot of PLS model based on NMR data from first two stages (red dots represent samples from stage I; blue dots represent samples from stage II). (b) PLS loading plot. Positive value corresponds to metabolites at higher concentration in the urine sample of stage I and vice versa.

**Figure 5:**
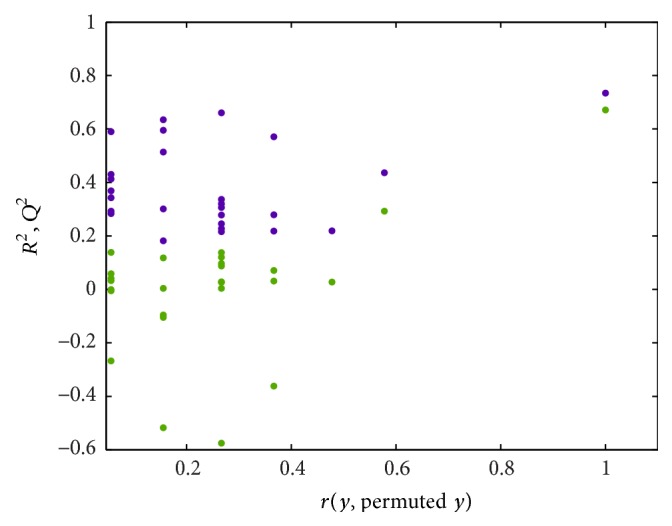
Permutation test of PLS model based on NMR data of first two stages.

A representative ^1^H NMR spectrum of a urine sample from an athlete is shown in Figure 2. Peaks of major metabolites could be observed in the spectra and were identified by comparing with a database [22] and data reported in the literature [6, 23]. With the urine samples, the PCA model was used to observe the distribution and the separation trends (Figure 3). A clear separation was found in the score plot of the PCA model based on the first two stages. To enlarge the difference and detect important variables, the PLS-DA model was established using one predictive component with *R* ^2^ = 0.734 and *Q* ^2^ = 0.671, as shown in Figure 4(a). The model was validated by a permutation test (Figure 5). As Figure 4 showed, *Q* ^2^ value of model was much higher than any other *Q* ^2^ from permutated model. This result indicated that the model with satisfied predictability was not overfitted. Since the PCA model of NMR data from stages two and three did not show clear separation (Figure 3), PLS was applied with *Q* ^2^ = 0.288, which indicates an unsatisfied model with low predictive ability.  Figure 4(b) displays a loading plot of the PLS-DA model from the first two stages. The loading plot illustrates the trends of major metabolite levels between the two groups. Concentrations of major metabolites, including TMAO, taurine, and hippurate, were higher in urine samples from the relatively higher training intensity and amount group, whereas creatinine, glycine, and an unknown signal at 3.68 ppm, which falls in the spectral region of sugars, polyols, and amino acid CH-groups, were obviously higher in the first stage group. To validate this trend and explore other metabolites that contribute to variations between the two groups, an integral value of identified metabolites was compared using the significance test. Decreased urinary lactate, alanine, trimethylamine, malonate, taurine, and glycine levels, and increased urinary TMAO and phenylalanine levels, were observed in samples from the higher training intensity and amount stage (Table 2). These urinary metabolites with statistically significant levels could be used as potential biomarkers contributing to metabolic changes in athletes in different physical states. In addition to those mentioned above, other metabolites, such as succinate, dimethylamine, creatinine, and an unknown signal in the region of 3.5–3.7 ppm, were close to the margin of statistical significance. The levels of these metabolites were decreased in urine samples from stages one to two. The observed trends from the significance test were in agreement with those from the PLS loading plot.

### 3.3. Hierarchical Cluster Analysis

**Figure 6:**
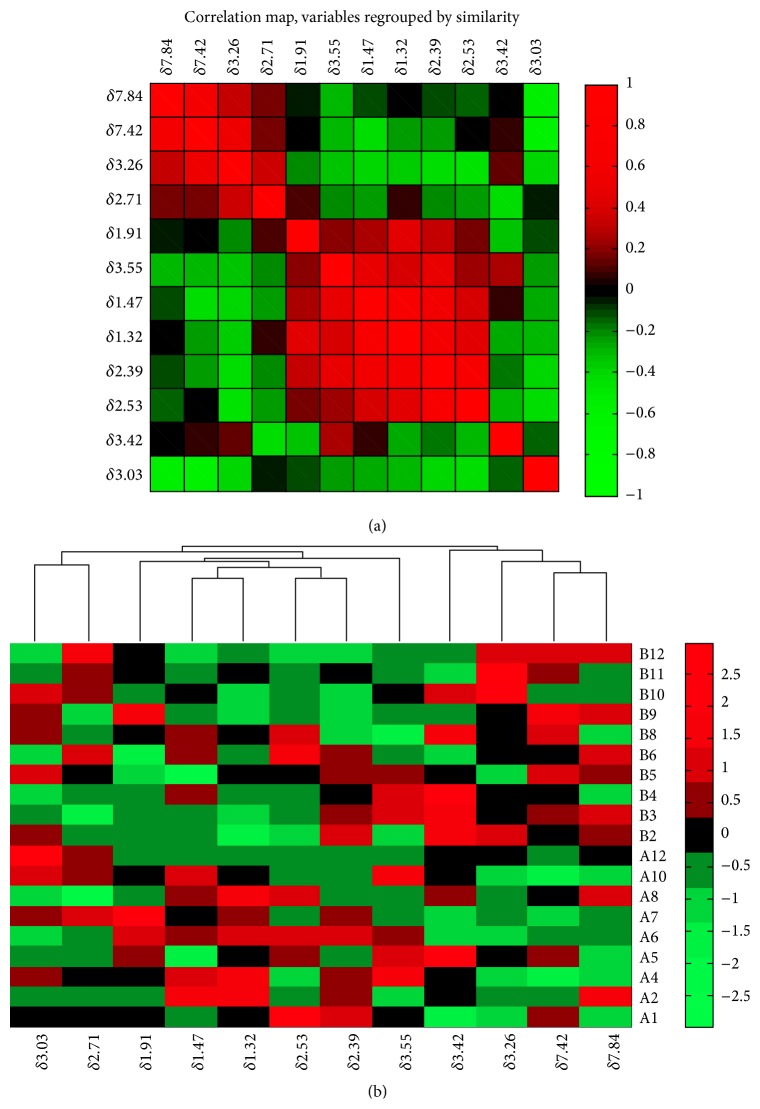
Correlation map (a) of the identified metabolites in stage I and stage II. Heat map (b) for identified metabolites in samples from first two stages (group A: first training stage; group B: second training stage). The upper dendrogram showed hierarchical clustering of variances corresponding to column data in the heat map. Rows: samples; columns: chemical shift of significant signal of metabolites.

To determine the relationships among major metabolites, correlation analysis and hierarchical clustering analysis were performed on NMR data and illustrated by a dendrogram and heat map (Figure 6). The correlation structure was conveniently shown through different colors in correlation map. Metabolites that highly correlate should cluster together in the dendrogram. Three clusters were clearly observed: (1) dimethylamine (*δ*2.71) and creatinine (*δ*3.03); (2) acetate (*δ*1.91), alanine (*δ*1.47), lactate (*δ*1.32), citrate (*δ*2.53), succinate (*δ*2.39), and glycine (*δ*3.55); and (3) taurine (*δ*3.42), TMAO (*δ*3.26), phenylalanine (*δ*7.42), and hippurate (*δ*7.84).

## 4. Discussion

As a routine examination, blood test was accomplished each week through the entire training program. During this period, hormone concentrations in serum, such as testosterone and cortisol, did not show significant variance because of one-day rest before sampling. This result is in accordance with a previous study [21]. As a sensitive indicator of training, the average value of creatine kinase concentration of first-stage samples was 620.3 ± 1007.7 U/L, which is much higher than the other two sample sets. By inspecting the original data (298, 228, 332, 125, 338, 171, 60, 745, 1011, 83, 3842, and 210 U/L), three values are out of the normal range (<400 U/L). However, the abnormal creatine kinase values returned to normal in the next training stage, which illustrates adaptation to training from the so-called repeated-bout effect [24]. Mononuclear cell counts decreased markedly at first but then increased along with the amount reduction of training, whereas other leukocytes (lymphocytes and neutrophils) did not show significant changes during training. The normal range of hemoglobin values was different between male and female samples, indicating that it depends on gender. The significant variations in the hemoglobin values in male samples suggest a reasonable training program for the athletes.

In this study, the urinary metabolic profiles of professional half-pipe snowboarders were investigated and multivariate analysis was performed on the datasets to detect variations in metabolites that are caused by changes in the amount and intensity of training. After performing high-throughput metabolomics analyses, the dynamic metabolic profiles of urine samples from the athletes were recorded. Variation between the first two training stages was clearly observed, which reflects changes of physical states of the athletes. In contrast, differences in the metabolic profiles between the last two training stages were not significant based on the PLS model.

**Figure 7:**
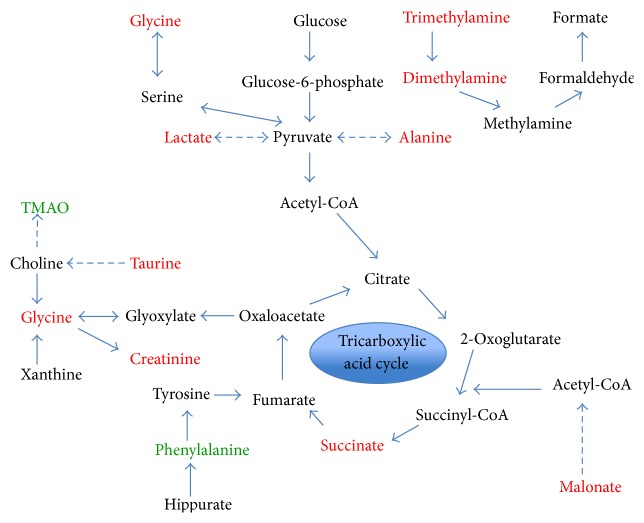
Summary of the metabolic pathways with indication of the effect of first two weeks of training on urine metabolite levels. Color coding: black, no change; red, upregulation; green, downregulation.

By analyzing the differential metabolites caused by training level, they were related to each other through metabolic pathways and are depicted in Figure 7. The tricarboxylic acid (TCA) cycle was considered an important pathway related to energy generation in exercise science research [25]. As an intermediate in the TCA cycle, the decreased levels of succinate between stages one and two indicate that the TCA cycle could be affected by long-term training based on different exercise loads.

Based on the heat map in Figure 6, lactate (peak at 1.32 ppm) exhibits a positive correlation with alanine (peak at 1.47 ppm). This result is in accordance with expectation because both of them served as substrates in gluconeogenesis [26, 27]. The concentration of creatinine also showed a distinct trend toward significance in the PLS model. It displayed a decreased level after one week training of higher level compared to the initial week. From here we could presume that the storage ability of phosphagen in muscle was improved, and consequently the level of excreted creatinine decreased after the second stage.

Trimethylamine levels in urine samples decreased in the second training stage. In contrast, the level of trimethylamine-*N*-oxide (TMAO) which is synthesized from endogenous trimethylamine increased. Because TMAO level could be elevated by consuming seafood which was included in the daily diet, the increased level of TMAO in urine samples may be due to increased food intake with high physical consumption during the second training stage. TMAO concentration also displayed remarkable variations in urine samples collected before and after exercise in some studies [6, 17, 18]. Thus, regulation of one-week training could be another reason why concentration level changed.

In addition, hippurate and its precursor phenylalanine [28] displayed a high correlation in hierarchical cluster analysis. As an essential amino acid, phenylalanine plays an important role in the synthesis of neurotransmitters. The increased level indicates metabolic adaption in the athlete as a response to the increased training load.

## 5. Conclusion

The present study evaluated changes in the urinary metabolic profiles of half-pipe snowboarders as a result of training, using a metabolomics strategy. The PCA score plot showed the tendency to separate athletes with different training amounts and intensities. Furthermore, PLS model was established to explore the differential metabolites caused by internal metabolic changes. These results proved the perturbations of related pathway and demonstrated that organisms reach a relatively stable physical state to adapt to the training load after long-term training. The study of metabolic adaption to different training level could help reveal the influence of long-term exercise on physiological status and provide useful information for formulating training programs.

## Supplementary Material

The Supplementary material contains 2D NMR spectrum of representative sample for the purpose of confirming the identification of metabolites in the text.
Supplementary Figure 1S is DQF-COSY NMR spectrum for typical urine sample which was used to observe ^1^H-^1^H correlation in compound.
Supplementary Figure 2S is HSQC NMR spectrum (Part I) for typical urine sample which was used to observe directly-bonded ^1^H and ^13^C correlation.
Supplementary Figure 3S contains HSQC NMR spectrum (Part II) for typical urine sample (Part II).

## Figures and Tables

**Table 1:** Clinical indicators and blood cell levels in different stages.

	Stage I	Stage II	Stage III	*P*_I,II_ value	*P*_II,III_ value
T (ng/dL)					
M	571.4 ± 173.0	557.8 ± 176.5	600.0 ± 148.8	0.68	0.41
F	48.6 ± 13.9	45.3 ± 6.9	44.1 ± 11.1	0.48	0.75
C (*μ*g/dl)	17.8 ± 2.5	17.01 ± 2.98	17.94 ± 4.5	0.11	0.50
CK (U/L)	620.3 ± 1007.7	163.6 ± 111.0	153.6 ± 131.3	0.14	0.84
BUN (mmol/L)	3.8 ± 0.8	4.0 ± 1.0	3.6 ± 0.7	0.49	0.08
WBC (*∗*10 · *e*3/*μ*L)	5.3 ± 0.9	5.2 ± 1.0	5.4 ± 1.2	0.71	0.60
LYM (*∗*10 · *e*3/*μ*L)	2.3 ± 0.5	2.3 ± 0.7	2.2 ± 0.7	0.83	0.73
MXD (*∗*10 · *e*3/*μ*L)	0.5 ± 0.1	0.3 ± 0.1	0.4 ± 0.2	0.01	0.20
NEUT (*∗*10 · *e*3/*μ*L)	2.6 ± 0.5	2.7 ± 0.7	2.8 ± 0.8	0.90	0.72
RBC (*∗*10 · *e*6/*μ*L)	4.7 ± 0.5	4.9 ± 0.7	4.8 ± 0.5	0.02	0.31
HGB (g/L)					
M	152.4 ± 6.3	160.8 ± 9.1	149.0 ± 6.6	0.03	0.03
F	129.6 ± 7.7	129.0 ± 8.8	132.6 ± 12.1	0.77	0.13
HCT	0.42 ± 0.04	0.43 ± 0.05	0.43 ± 0.04	0.07	0.57
MCV (*μ*m^3^)	89.3 ± 2.4	88.3 ± 2.6	89.3 ± 2.8	2.6 *∗* 10^−3^	5 *∗* 10^−3^
PLT (*∗*10 · *e*3/*μ*L)	226.6 ± 47.5	165.0 ± 34.2	223.8 ± 38.4	1.5 *∗* 10^−4^	3.65 *∗* 10^−5^

Values are given as the means ± SD.

T: testosterone; C: cortisol; CK: creatine kinase; BUN: blood urea nitrogen; WBC: white blood cells; LYM: lymphocyte count; MXD: mononuclear cell count; NEUT: neutrophil count; RBC: red blood cells; HGB: hemoglobin; HCT: hematocrit; MCV: mean corpuscular volume; PLT: platelet; M: male; F: female.

**Table 2:** List of peaks intensity with *P* < 0.05 from urinary spectra of first two stages.

Number	*δ* (ppm)	Metabolite	Trend	Significance
1	1.32	Lactate	↓	*∗*
2	1.47	Alanine	↓	*∗*
3	2.91	Trimethylamine	↓	*∗∗*
4	3.14	Malonate	↓	*∗*
5	3.26	TMAO	↑	*∗∗*
6	3.42	Taurine	↓	*∗∗*
7	3.55	Glycine	↓	*∗*
8	7.42	Phenylalanine	↑	*∗*

The potential biomarkers were labeled with downregulation (↓) and upregulation (↑). ^*∗*^ *P* < 0.05 and ^*∗∗*^ *P* < 0.01.

## References

[1] Smith L. L. (2000). Cytokine hypothesis of overtraining: a physiological adaptation to excessive stress?. *Medicine and Science in Sports and Exercise*.

[2] Smith D. J., Roberts D. (1994). Effects of high volume and/or intense exercise on selected blood chemistry parameters. *Clinical Biochemistry*.

[3] Yan B., Jiye A., Wang G. (2009). Metabolomic investigation into variation of endogenous metabolites in professional athletes subject to strength-endurance training. *Journal of Applied Physiology*.

[4] Margonis K., Fatouros I. G., Jamurtas A. Z. (2007). Oxidative stress biomarkers responses to physical overtraining: implications for diagnosis. *Free Radical Biology and Medicine*.

[5] Nicholson J. K., Lindon J. C. (2008). Systems biology: metabonomics. *Nature*.

[6] Pechlivanis A., Kostidis S., Saraslanidis P. (2010). H-1 NMR-based metabonomic investigation of the effect of two different exercise sessions on the metabolic fingerprint of human urine. *Journal of Proteome Research*.

[7] Santone C., Dinallod V., Paci M., D'Ottavio S., Barbato G., Bernardini S. (2014). Saliva metabolomics by NMR for the evaluation of sport performance. *Journal of Pharmaceutical and Biomedical Analysis*.

[8] Nicholson J. K., Lindon J. C., Holmes E. (1999). ‘Metabonomics’: understanding the metabolic responses of living systems to pathophysiological stimuli via multivariate statistical analysis of biological NMR spectroscopic data. *Xenobiotica*.

[9] Weljie A. M., Jirik F. R. (2011). Hypoxia-induced metabolic shifts in cancer cells: moving beyond the Warburg effect. *International Journal of Biochemistry and Cell Biology*.

[10] Lindon J. C., Holmes E., Bollard M. E., Stanley E. G., Nicholson J. K. (2004). Metabonomics technologies and their applications in physiological monitoring, drug safety assessment and disease diagnosis. *Biomarkers*.

[11] Dumas M.-E., Maibaum E. C., Teague C. (2006). Assessment of analytical reproducibility of 1H NMR spectroscopy based metabonomics for large-scale epidemiological research: the INTERMAP study. *Analytical Chemistry*.

[12] Pohjanen E., Thysell E., Jonsson P. (2007). A multivariate screening strategy for investigating metabolic effects of strenuous physical exercise in human serum. *Journal of Proteome Research*.

[13] Kirwan G. M., Coffey V. G., Niere J. O., Hawley J. A., Adams M. J. (2009). Spectroscopic correlation analysis of NMR-based metabonomics in exercise science. *Analytica Chimica Acta*.

[14] Hodgson A. B., Randell R. K., Boon N. (2013). Metabolic response to green tea extract during rest and moderate-intensity exercise. *Journal of Nutritional Biochemistry*.

[15] Chorell E., Moritz T., Branth S., Antti H., Svensson M. B. (2009). Predictive metabolomics evaluation of nutrition-modulated metabolic stress responses in human blood serum during the early recovery phase of strenuous physical exercise. *Journal of Proteome Research*.

[16] Bruce S. J., Breton I., Decombaz J. (2010). A plasma global metabolic profiling approach applied to an exercise study monitoring the effects of glucose, galactose and fructose drinks during post-exercise recovery. *Journal of Chromatography B: Analytical Technologies in the Biomedical and Life Sciences*.

[17] Miccheli A., Marini F., Capuani G. (2009). The influence of a sports drink on the postexercise metabolism of elite athletes as investigated by nmr-based metabolomics. *Journal of the American College of Nutrition*.

[18] Enea C., Seguin F., Petitpas-Mulliez J. (2010). ^1^H NMR-based metabolomics approach for exploring urinary metabolome modifications after acute and chronic physical exercise. *Analytical and Bioanalytical Chemistry*.

[19] Papacosta E., Nassis G. P. (2011). Saliva as a tool for monitoring steroid, peptide and immune markers in sport and exercise science. *Journal of Science and Medicine in Sport*.

[20] Howard R., Carriquiry A. L., Beavis W. D. (2014). Parametric and nonparametric statistical methods for genomic selection of traits with additive and epistatic genetic architectures. *G3-Genes Genomes Genetics*.

[21] Eisen M. B., Spellman P. T., Brown P. O., Botstein D. (1998). Cluster analysis and display of genome-wide expression patterns. *Proceedings of the National Academy of Sciences of the United States of America*.

[22] Wishart D. S., Tzur D., Knox C. (2007). HMDB: the human metabolome database. *Nucleic Acids Research*.

[23] Rasmussen L. G., Savorani F., Larsen T. M., Dragsted L. O., Astrup A., Engelsen S. B. (2011). Standardization of factors that influence human urine metabolomics. *Metabolomics*.

[24] McHugh M. P. (2003). Recent advances in the understanding of the repeated bout effect: the protective effect against muscle damage from a single bout of eccentric exercise. *Scandinavian Journal of Medicine & Science in Sports*.

[25] Howarth K. R., LeBlanc P. J., Heigenhauser G. J. F., Gibala M. J. (2004). Effect of endurance training on muscle TCA cycle metabolism during exercise in humans. *Journal of Applied Physiology*.

[26] Perriello G., Jorde R., Nurjhan N. (1995). Estimation of glucose-alanine-lactate-glutamine cycles in postabsorptive humans: role of skeletal muscle. *The American Journal of Physiology—Endocrinology and Metabolism*.

[27] Tang F.-C. (2006). Influence of branched-chain amino acid supplementation on urinary protein metabolite concentrations after swimming. *Journal of the American College of Nutrition*.

[28] Lees H. J., Swann J. R., Wilson I. D., Nicholson J. K., Holmes E. (2013). Hippurate: the natural history of a mammalian-microbial cometabolite. *Journal of Proteome Research*.

