# Supplementary material for: Applying 1H NMR Spectroscopy to Detect Changes in the Urinary Metabolite Levels of Chinese Half-Pipe Snowboarders after Different Exercises
Source: J Anal Methods Chem. 2015 May 25;2015:315217. doi: 10.1155/2015/315217 (PMC4458538; doi:10.1155/2015/315217)
Supplement: Supplementary file 1 — The Supplementary material contains 2D NMR spectrum of representative sample for the purpose of confirming the identification of metabolites in the text. Supplementary Figure 1S is DQF-COSY NMR spectrum for typical urine sample which was used to observe 1H-1H correlation in compound. Supplementary Figure 2S is HSQC NMR spectrum (Part I) for typical urine sample which was used to observe directly-bonded 1H and 13C correlation. Supplementary Figure 3S contains HSQC NMR spectrum (Part II) for typical urine sample (Part II). [file 315217.f1.pdf]

Applying ^{1}H NMR Spectroscopy to detect Changes in the Urinary Metabolite Levels of Chinese Halfpipe Snowboarders after Different Exercises

Fuqiu Wang, [1] Jiao Han, [2] Qing He,[3] Zhufeng Geng,[2] Zhiwei Deng,[2] and Decai Qiao[1]

[1] *College of P.E and Sports, Beijing Normal University, No.19 Xinjiekouwai Street, Haidian District, Beijing 100875, China*

[2] *Center of Analysis and Test, Beijing Normal University, No.19 Xinjiekouwai Street, Haidian District, Beijing 100875, China*

[3] *School of Chemical Engineering and Technology, Tianjin University, 92 Weijin Road, Nankai District,Tianjin 300072, China*

* Corresponding authors: Decai Qiao, E-Mail: decaiq@bnu.edu.cn, Tel.: +86-10-5880-2227, Fax: +86-10-5880-2227 and Zhiwei Deng, E-Mail: dengzw@bnu.edu.cn , Tel.: +86-10-5880-6866; Fax: +86-10-5880-0076.

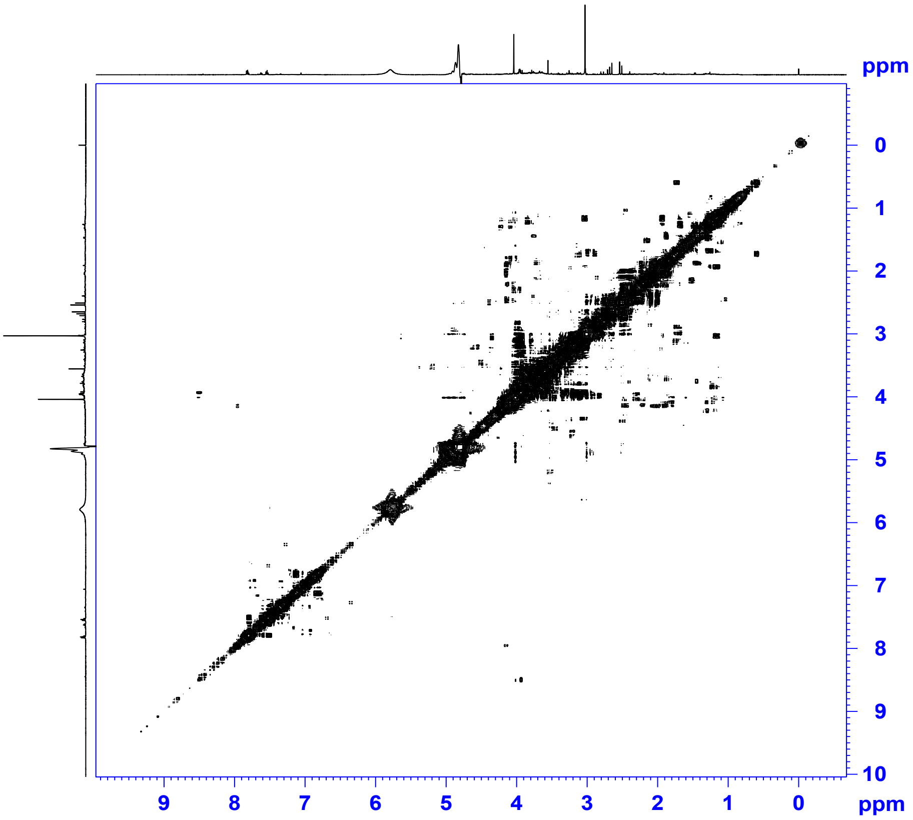

Figure 1S COSY NMR spectrum for typical urine sample.

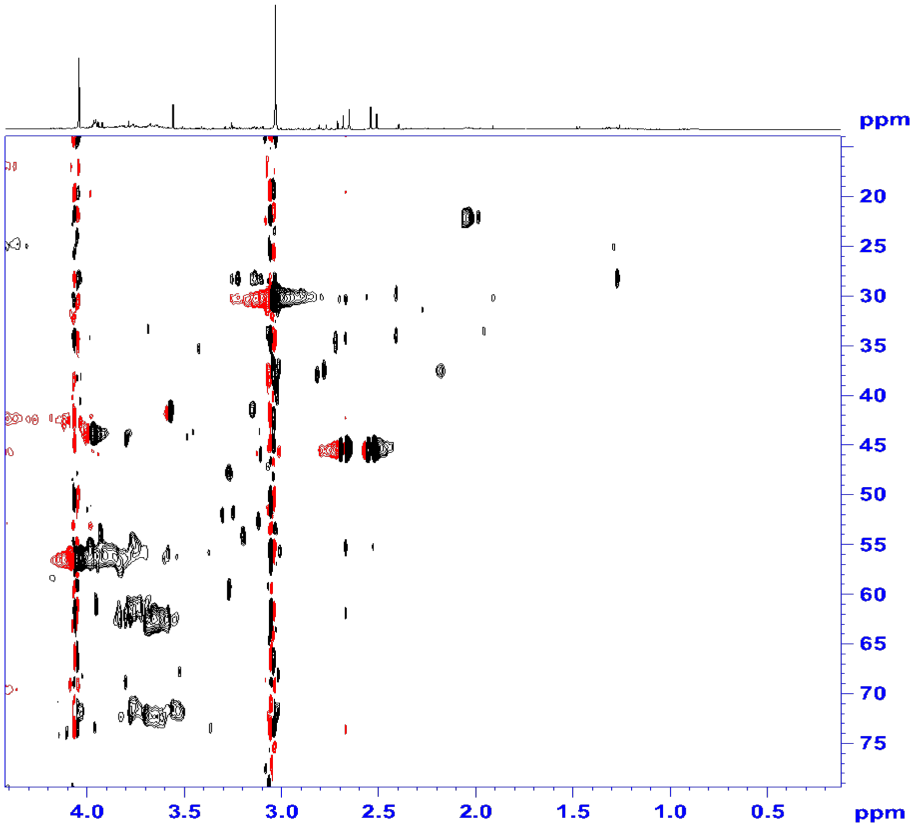

Figure 2S HSQC NMR spectrum for typical urine sample (Part I)

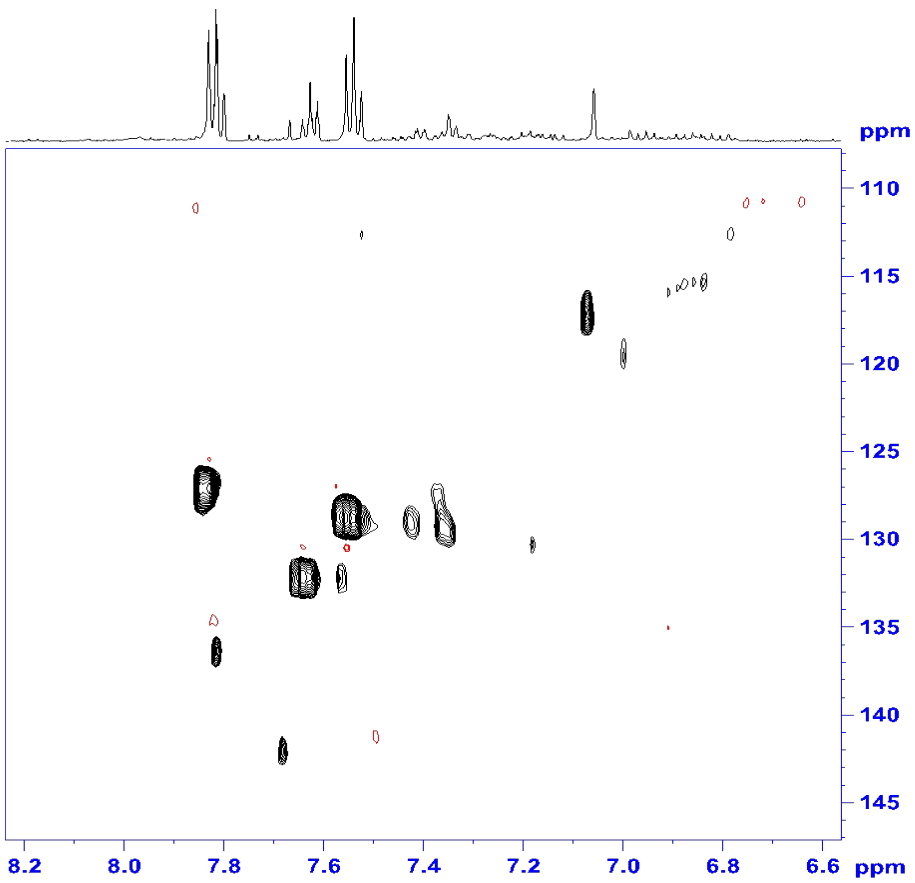

Figure 3S HSQC NMR spectrum for typical urine sample (Part II)
